# [Retracted] Salinomycin triggers prostate cancer cell apoptosis by inducing oxidative and endoplasmic reticulum stress via suppressing Nrf2 signaling 

**DOI:** 10.3892/etm.2026.13166

**Published:** 2026-04-20

**Authors:** Jianyong Yu, Yang Yang, Shan Li, Peng Meng

Exp Ther Med 22:546, 2021; DOI: 10.3892/etm.2021.10378

Following the publication of the above paper, the authors requested that a corrigendum be published for Fig. 5 on account of the fact that the gel bands selected for Nrf2 and GAPDH (in Fig. 5A) had been wrongly used. Upon performing an independent analysis of the data in this paper in the Editorial Office, it also came to light that the protein band for PERK in Fig. 3A for the 5 μM salinomycin experiment with the PC-3 cell line was strikingly similar to the band shown for the 0 μM salinomycin experiment for the DU145 cell line. Furthermore, in Fig. 4A, the protein bands for HO-1 for the 2 and 5 μM salinomycin experiments for the PC-3 and DU145 cell lines also appeared to be broadly similar.

Upon asking the authors to provide an explanation for these additional figures, they were able to show how Figs. 3 and 5 had been erroneously assembled, but were not able to retrieve the complete uncropped exposure files for the data in Fig. 4A owing to data archiving limitations at the time. Although alternative data were offered for the corrigendum for Fig. 4, following this internal enquiry the Editor of *Experimental and Therapeutic Medicine* has decided that this paper should be retracted due to an overall lack of confidence in the presentation of Fig. 4 as it was originally shown. After contacting the authors, they accepted the decision to retract the paper. The Editor apologizes to the readership of the Journal for any inconvenience caused.

